# Effects of Acute Moderate- and High-Intensity Aerobic Exercise on Oxygenation in Prefrontal Cortex of Male Methamphetamine-Dependent Patients

**DOI:** 10.3389/fpsyg.2022.801531

**Published:** 2022-01-26

**Authors:** Siyu Gao, Chenglin Zhou, Yifan Chen

**Affiliations:** ^1^Department of Psychology, Shanghai University of Sport, Shanghai, China; ^2^Department of Physical Education and Humanities, Nanjing Sport Institute, Nanjing, China

**Keywords:** acute aerobic exercise, methamphetamine dependence, fNIRS, prefrontal cortex, functional connectivity

## Abstract

The aim of this study was to explore the influence of different intensities of acute aerobic exercise on brain activation in male methamphetamine (MA)-dependent patients during exercise. Twenty MA-dependent patients were divided randomly into two groups participating in 35 min of either moderate- or high-intensity aerobic exercise. Functional near-infrared spectral imaging (fNIRS) was used to detect hemodynamic changes in prefrontal cortex during the main 25-min exercise stage. The results revealed that high-intensity acute aerobic exercise aroused more cerebral oxygenation changes in the prefrontal cortex and left dorsolateral prefrontal cortex during exercise as compared with moderate-intensity exercise. Furthermore, there was a stronger positive connection observed between orbital frontal cortex and left dorsolateral prefrontal cortex in the high-intensity group than in the moderate-intensity group. Together these results suggest that for submaximal exercise intensities, high-intensity exercise may bring more benefits to male MA-dependent patients than moderate-intensity.

## Introduction

Exercise program, especially aerobic exercise has been considered to be an effective treatment for drug abuse (Abrantes and Blevins, 2019). Previous studies demonstrated that it could relieve symptoms caused by drug abuse through various avenues: exercise-induced neuroplasticity in the prefrontal cortex (PFC) may enhance executive function and it is helpful for decreasing compulsive behaviors such as drug seeking (Costa et al., 2019); exercise may modulate brain dopamine levels via epigenetic interactions with brain-derived neurotrophic factor to heighten reward sensitivity and restore functioning of the reward system (Nock et al., 2017); exercise can also reduce negative emotions associated with depression and anxiety (Ashdown-Franks et al., 2020).

Although a large number of relevant studies have shown positive effects of aerobic exercise on drug addicts, the neurological mechanisms remain unclear. Changes in the levels of oxygenated and deoxygenated hemoglobin in PFC that accompany exercise offer a possible neural basis (Giles et al., 2014). Increased brain oxygenation during and after exercise is also a potential mechanism for explaining cognitive improvement (Jung et al., 2015). Researchers have used functional near-infrared spectral imaging (fNIRS) to demonstrate that the change of cortical cerebral blood flow in normal people during aerobic exercise will increase with increasing exercise intensity, especially in PFC (Rooks et al., 2010); and, for submaximal exercise intensities, increasing intensity promotes cerebral oxygenation of PFC (Subudhi et al., 2009; Giles et al., 2014). However, there has been very little direct research into how PFC activation of drug-dependent patients is affected by aerobic exercise. In those drug-dependent patients with damaged PFC, whether there is a positive relationship between exercise intensity and exercise effect still needs to be clarified by empirical research.

Thus, this study aimed to explore the influence of different intensities of acute aerobic exercise on the activation of PFC in male methamphetamine (MA)-dependent patients. A fNIRS measure was used to detect hemodynamic changes in PFC during exercise. The effects of exercise of different intensities were systematically compared to explore whether there is still a positive relationship between intensity and exercise effect in MA-dependent patients.

## Materials and Methods

### Participants

Twenty male participants aged 18–45 years were recruited from the Chinese Shiliping Drug Rehabilitation Bureau in Zhejiang. All subjects met the DSM-V criteria for MA-dependent according to the Structured Diagnostic Interview, and has abstinent for at least 3 months. The exclusion criteria were a history of psychosis, physical or medical conditions that contraindicated exercise. Then, the subjects were divided randomly into two groups: moderate- and high-intensity exercise groups. The demographic characteristics of two groups are shown in Table 1. All subjects were informed about the study and provided informed consent. All the procedures adhered to the ethical guidelines of the Declaration of Helsinki and were approved by the ethics committee of Shanghai University of Sport (102772019RT044).

**TABLE 1:** Subjects’ demographic characteristics (M ± SE).

	High-intensity group (*n* = 10)	Moderate-intensity group (*n* = 10)
Age (year)	31.30 ± 0.97	32.70 ± 0.58
Number of relapse (time/year)	2.40 ± 0.34	1.90 ± 0.28
Duration of drug use (year)	3.90 ± 0.38	5.20 ± 0.66
Weight (kg)	70.82 ± 3.36	71.57 ± 2.84
Height (m)	1.72 ± 0.01	1.70 ± 0.01
BMI	23.92 ± 0.98	24.72 ± 0.70
Duration of education (year)	9.00 ± 0.00	8.40 ± 0.75

*Number of relapse means the mean relapse time per year before abstinence.*

*No significant difference in any characteristic was detected among groups (all p > 0.05).*

### Acute Aerobic Exercise Intervention

Subjects in the two exercise groups were engaged in exercise programs that involved a 35-min exercise program by using cycle ergometer at moderate and high intensity, respectively, which was created in accordance with the recommendations of the American College of Sports Medicine (Ferguson, 2014). The intervention involved warm-up (5 min), aerobic exercise as the main exercise stage (25 min), and cool-down for 5 min. The moderate-intensity group needed to reach 65–75% of the indirect maximum heart rate (HRmax) during main exercise stage, and the high-intensity group needed to reach 76–85% of the HRmax. HRmax values were calculated as 206.9–0.67 × age. During the program, HR of the subjects was monitored by Smart Sensors (Suunto, Vantaa, Finland). All interventions were performed at the drug rehabilitation bureau.

### Functional Near-Infrared Spectral Imaging Data Acquisition and Processing

**FIGURE 1:**
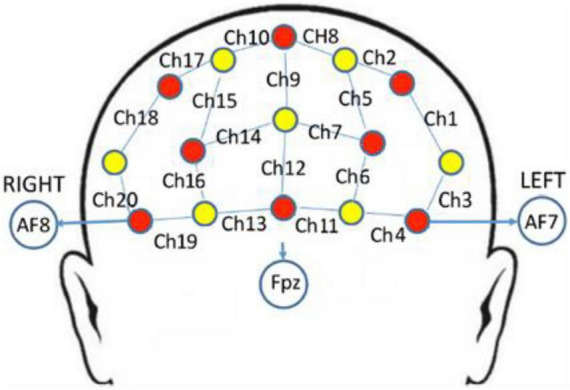
fNIRS channels mapped on the brain. One source (red circles) and one detector (yellow circles) formed a channel (blue lines), and 20 channels were formed (numbered in black, i.e., Ch1).

A multi-channel continuous-wave fNIRS instrument (NIRScout; NIRx Medical Technologies LLC, Minneapolis, MN, United States) was used to monitor hemodynamic activity during the 25-min main exercise stage. The fNIRS probe consisted of eight dual-wavelength sources (780 and 830 nm) and seven optical detectors. The sampling rate was 7.81 Hz. The probes were arranged in accordance with the 10–20 system, and each emitter was no more than 3 cm away from its corresponding detector. One source and one detector formed a channel, and 20 channels were formed in PFC (Figure 1). Information on the correspondence between fNIRS channel locations and specific brain regions was taken from Okamoto et al. (2004) and Tsuzuki et al. (2007). The locations of all channels in PFC were converted to standard Montreal Neurological Institute coordinates using a probabilistic estimation method (Cutini et al., 2011). Six regions of interest (ROIs) were formed (Table 2).

**TABLE 2:** Location for each channel.

Regions of interest (ROIs)	Hemisphere	Channels	MNI coordinates		
			X	Y	Z
Ventrolateral prefrontal cortex (VLPFC)	L	1_‵_3_‵_4_‵_	−41	52	−6
	R	18_‵_19_‵_20	41	51	−7
Dorsolateral prefrontal cortex (DLPFC)	L	2_‵_5_‵_7_‵_8_‵_	−20	45	43
	R	9_‵_10_‵_14_‵_15_‵_17	19	49	42
Front polar area (FPA)		6_‵_12_‵_16	0	65	25
Orbital frontal cortex (OFC)		11_‵_13	0	69	−1

*L, left; R, right.*

*MNI, Montreal Neurological Institute.*

**FIGURE 2:**
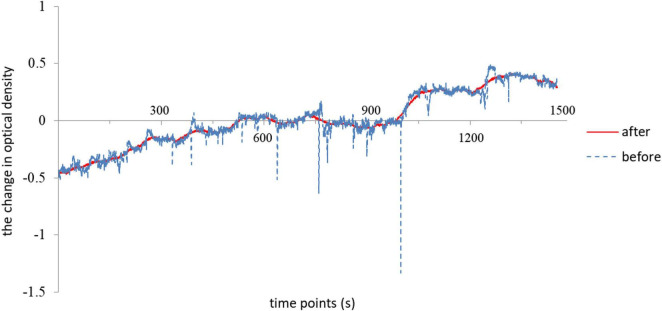
The time series data changes in one channel of one subject before and after artifacts removal by using channel-based cubic spline interpolation.

Data were analyzed using Homer2 open source software (MGH-Martinos Center for Biomedical Imaging, Boston, MA, United States), implemented in MATLAB using channel-based cubic spline interpolation was used to detect and remove motion artifacts (the time series data changes in one channel of one subject before and after artifacts removal was showed in Figure 2). Then, a 0.01-Hz high-pass filter and a 0.10-Hz low-pass filter were used to remove baseline drift and reduce the impact of physiological reactions such as heartbeat and blood pressure on the fNIRS signals. The modified Beer-Lambert law was applied to calculate hemoglobin concentration changes. The data were averaged from the time window 0--25 min to obtain an average response to the aerobic exercise for each channel in each participant. The concentration of the oxygenated hemoglobin ([HbO]) and deoxygenated hemoglobin signals ([Hb]) were chosen as the primary metrics. The [HbO] and [Hb] data of each ROI were averaged from the values of multiple channels it contains. Additionally, FC_NIRS^1^ was used to calculate Pearson’s correlation between the selected seed ROI and other ROIs to weigh the strength of functional connectivity of the pairwise relationships. Prior to any statistical analysis, the correlation values were converted to Fisher *z*-values using the Fisher z-transformation.

### Statistical Analysis

Data of one participant in each of the two groups was excluded because of excessive artifacts; the remaining 18 participants were included in the final analyses. The Mann-Whitney *U*-test was used to compare the difference between two groups and the Benjamini-Hochberg method was used to correct for multiple comparisons of variables for each ROI and time window by SPSS 20.0 software (SPSS Inc., Chicago, IL, United States). The comparison of mean [HbO] of each ROI during 25-min exercise vs. 0-value has been performed to test the existence of a group activation. Mean values for the behavioral variables were reported with standard errors. The statistical significance level was set to *p* < 0.05.

## Results

### Effects of Acute Aerobic Exercise on [HbO] and [Hb]

**FIGURE 3:**
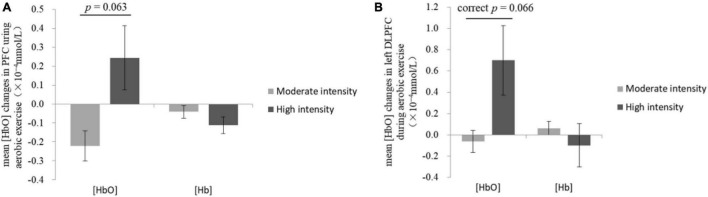
Changes of mean [HbO] and [Hb] in the prefrontal cortex **(A)** and the left dorsolateral prefrontal cortex **(B)** during exercise.

Mann-Whitney *U*-test showed that the mean [HbO] of PFC in the high-intensity group (0.24 × 10^–4^ ± 0.17 × 10^–4^ mmol/L) was higher than that of the moderate-intensity group (−0.22 × 10^–4^ ± 0.08 × 10^–4^ mmol/L; *Z* = −1.90, *p* = 0.063; Figure 3A).

**FIGURE 4:**
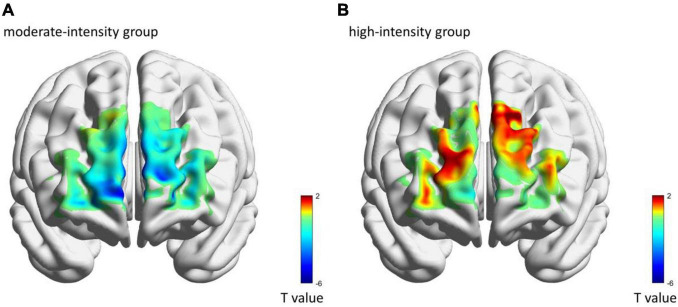
The average brain activation of each channel in moderate-intensity **(A)** and high-intensity **(B)** groups during 25-min exercise. Colors represent the *t*-value of mean [HbO] as compared with 0-value.

The comparisons of group differences in each ROI showed that only in left DLPFC, the mean [HbO] of the high-intensity group (0.70 × 10^–4^ ± 0.33 × 10^–4^ mmol/L) was higher than that of the moderate-intensity group (−0.06 × 10^–4^ ± 0.10 × 10^–4^ mmol/L; *Z* = −2.52, uncorrected *p* = 0.011, corrected *p* = 0.066; Figure 3B). Mean [Hb] values did not differ significantly between the two groups. No significant differences between groups were found in other ROIs. The average activation of PFC in the two groups during 25-min exercise is shown in Figure 4.

**FIGURE 5:**
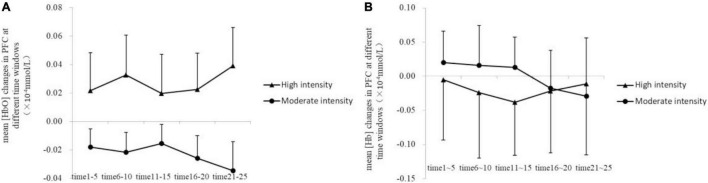
Changes of mean [HbO] **(A)** and [Hb] **(B)** in the prefrontal cortex at different time windows.

Further analysis of group differences at different time windows showed that only in time window 5 (21–25 min of exercise stage), the mean [HbO] of the high-intensity group in PFC (0.35 × 10^–4^ ± 0.27 × 10^–4^ mmol/L) was higher than that of the moderate-intensity group (−0.29 × 10^–4^ ± 0.20 × 10^–4^mmol/L; *Z* = −1.90, uncorrected *p* = 0.063, corrected *p* = 0.27; Figure 5). Mean [Hb] values did not differ significantly between the two groups.

### Effects of Acute Aerobic Exercise on Functional Connectivity

**FIGURE 6:**
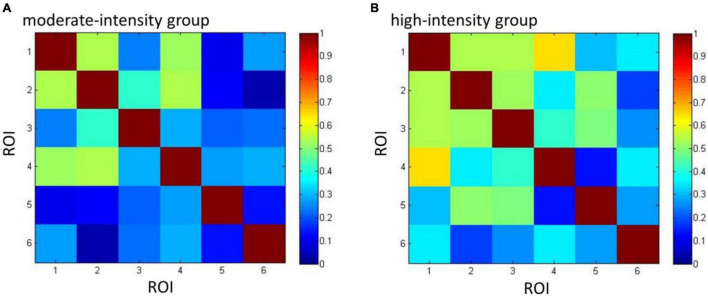
The connectivity matrices of moderate- **(A)** and high-intensity **(B)** groups. Numbers 1–6 represent the ROIs (1 = FPA, 2 = OFC, 3 = VLPFC_L, 4 = VLPFC_R, 5 = DLPFC_L, 6 = DLPFC_R) and colors represent the mean group-level Pearson’s correlation between each pair of ROIs.

Group connectivity matrices are shown in Figure 6. A group difference was observed in left DLPFC; we therefore used it as the seed ROI, from which the correlation values of other ROIs were calculated. Results showed the correlation between left DLPFC and OFC was higher in the high-intensity group than in the moderate-intensity group (*Z* = −2.08, uncorrected *p* = 0.040, corrected *p* = 0.20), indicating that high-intensity acute aerobic exercise may enhance the connection between left DLPFC and OFC. No significant group differences were found in correlation between left DLPFC and other 4 ROIs.

## Discussion

The current study used fNIRS to explore the effect of moderate- and high-intensity acute aerobic exercise on changes in PFC activity in MA-dependent patients. We found that compared with moderate-intensity, high-intensity acute aerobic exercise induces more cerebral oxygenation changes in PFC during exercise, indicating that there may be a positive relationship between exercise intensity and exercise effect in MA-dependent patients. This is in line with previously published studies on healthy volunteers which found that high-intensity exercise promotes cerebral oxygenation changes in PFC when exercise intensity remains submaximal (Giles et al., 2014).

Exercise is known to promote the growth of blood vessels in the brain and to increase blood flow in response to physiological and neuronal changes (Ogoh and Ainslie, 2009); the excessive supply of local blood flow can lead to increases in [HbO] (Perrey, 2008). In the present study, the increase of [HbO] in PFC in the high-intensity group revealed that high intensities may be accompanied by increased oxygen supply to the brain during exercise, and this augmented oxygenation has been previously shown to be sufficient to meet physiological and cognitive demands (Tempest et al., 2017). It has also been demonstrated that high-intensity exercise is more suitable than moderate- or low-intensity for increasing aerobic capacity, which is a main determinant of increasing functional ability and thereby survival (Rognmo et al., 2004). Therefore, even if the PFC were impaired due to drug abuse, it may be possible to repair the brain and improve cognition by increasing brain oxygenation through high-intensity exercise. Consistent with previous studies (Greenwood and Fleshner, 2011; Smith and Lynch, 2012), moderate-intensity aerobic exercise did not significantly improve hemodynamic changes in PFC of these patients. Other studies have also found that when healthy young people completed 30 min of aerobic exercise at either low, moderate, or high intensity (defined as 52, 68, and 84% of HRmax, respectively), the high-intensity group experienced more [HbO] changes (Giles et al., 2014), suggesting that higher intensity exercise is more beneficial. Moreover, the current study also found group differences in the activation of left DLPFC. Although it did not reach a significant level, compared with moderate-intensity, high-intensity acute aerobic exercise caused more brain oxygenation changes in the left DLPFC during exercise. DLPFC is composed of spatially selective neurons, participates in sensory processing and motor signal transmission (Goldman-Rakic, 1995), and is the highest cortical area involved in motor planning, organization, and regulation. Previous studies have also found that acute aerobic exercise could enhance regional activity in this area (Hillman et al., 2009; Steinberg et al., 2019).

Our functional connectivity analysis showed that compared with moderate-intensity exercise, the positive connection between OFC and left DLPFC in high-intensity group had a higher tendency before correction. OFC is involved in processing motivation-related information and guiding behavior within the addiction loop, and is also responsible for monitoring information relating to reward or punishment and behavioral inhibition (Everitt et al., 2007). People with impairments in OFC exhibit weakened inhibitory ability and often behave inappropriately due to their inability to judge rewards and punishments, and their tendency toward impulsive behavior and compulsive responses (Winstanley, 2007). Dysfunction in OFC and DLPFC is a main cause of drug abuse. A previous study found that the increase in drug craving of nicotine-dependent people is accompanied by an increase in OFC activation, whereas lower levels of craving correspond to activation of DLPFC (Kroczek et al., 2015). The enhanced functional connection between OFC and left DLPFC in our study indicates that these two areas have more simultaneous activities, which may help patients to initiate inhibitory activities and suppress drug craving when faced with drug-related cues. Although the trend of difference in functional connectivity was relatively weak and disappeared after correction, it could explain that high-intensity aerobic exercise may have positive effect on the enhancement of brain function in MA-dependent patients to a certain extent, and it is hoped that such findings can be obvious by collecting more samples.

The current study has several limitations. Due to the particularity of the subjects there are restrictions on their recruitment, which leads to a slightly insufficient number of subjects participating in the experiment and some experimental results only showing marginal significance. Similarly, this study was limited by environmental conditions that only recruited male subjects and had a lack of female subjects. Previously published findings in healthy volunteers showed that overly high intensities may affect the efficiency of exercise interventions for cognitive function in female individuals, while male individuals can expect further improvements from highly intense programs (Ludyga et al., 2020). Therefore, it is essential to add female subjects in further studies to comprehensively investigate the complex relationship between exercise intensity and exercise effect in MA-dependent patients. Additionally, we only monitored participants’ HR to control exercise intensity without recording it. In future study, we should record HR data and analyze the correlations between it and brain signals to provide further evidence.

## Conclusion

The present study used the measurement of hemodynamic changes in PFC during acute aerobic exercise in MA-dependent subjects to demonstrate that high-intensity exercise induces more brain activation in PFC and more correlated activity between left DLPFC and OFC. Those results remind us that for submaximal exercise intensities, higher intensities may bring more benefits to male MA-dependent patients, such as the enhancement of brain activation.

## Data Availability Statement

The raw data supporting the conclusions of this article will be made available by the authors, without undue reservation.

## Ethics Statement

The studies involving human participants were reviewed and approved by the Ethics Committee, Shanghai University of Sport, Shanghai, China (102772019RT044). The patients/participants provided their written informed consent to participate in this study.

## Author Contributions

YC designed the experiment and gave final approval. SG and CZ conducted the experiment. SG and YC analyzed the data and drafted the manuscript. All authors contributed to the article and approved the submitted version.

## Conflict of Interest

The authors declare that the research was conducted in the absence of any commercial or financial relationships that could be construed as a potential conflict of interest.

## Publisher’s Note

All claims expressed in this article are solely those of the authors and do not necessarily represent those of their affiliated organizations, or those of the publisher, the editors and the reviewers. Any product that may be evaluated in this article, or claim that may be made by its manufacturer, is not guaranteed or endorsed by the publisher.

## Footnotes

1
https://www.nitrc.org/projects/fcnirs/
